# (2*R*)-2-(1,3-Dioxoisoindolin-2-yl)-3-methyl­butanoic acid

**DOI:** 10.1107/S1600536811033393

**Published:** 2011-08-27

**Authors:** Abdul Rauf Raza, Aisha Saddiqa, M. Nawaz Tahir, Sadia Saddiq

**Affiliations:** a Department of Chemistry, University of Sargodha, Sargodha, Pakistan; b Department of Physics, University of Sargodha, Sargodha, Pakistan

## Abstract

In the title compound, C_13_H_13_NO_4_, the dihedral angle between the nine-membered phthalimino ring system and the carb­oxy­lic acid group is 67.15 (9)°. An intra­molecular C—H⋯O close contact, which forms an *S*(6) ring, may help to establish the mol­ecular conformation. In the crystal, mol­ecules are linked by O—H⋯O hydrogen bonds, thereby forming *C*(7) chains propagating in [010].

## Related literature

For related structures, see: Barooah *et al.* (2006 ▶); Raza *et al.* (2009 ▶). For graph-set notation, see: Bernstein *et al.* (1995 ▶).
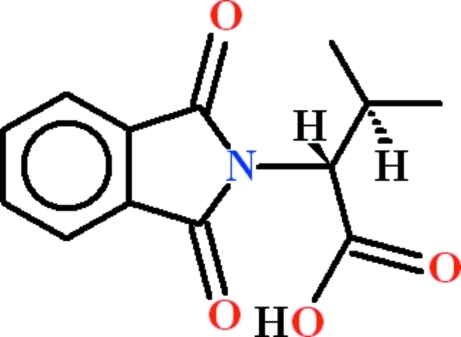


## Experimental

### 

#### Crystal data

C_13_H_13_NO_4_
*M* *_r_* = 247.24
Monoclinic, 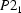

*a* = 8.9120 (7) Å
*b* = 6.3410 (4) Å
*c* = 11.8471 (10) Å
β = 109.980 (4)°
*V* = 629.20 (8) Å^3^
*Z* = 2
Mo *K*α radiation
μ = 0.10 mm^−1^
*T* = 296 K
0.34 × 0.26 × 0.24 mm

#### Data collection

Bruker Kappa APEXII CCD diffractometer
Absorption correction: multi-scan (*SADABS*; Bruker, 2005 ▶) *T*_min_ = 0.968, *T*_max_ = 0.978
5969 measured reflections
1635 independent reflections
1267 reflections with *I* > 2σ(*I*)
*R*_int_ = 0.027

#### Refinement

*R*[*F* ^2^ > 2σ(*F* ^2^)] = 0.041
*wR*(*F* ^2^) = 0.100
*S* = 1.05
1635 reflections
166 parameters
H-atom parameters constrained
Δρ_max_ = 0.13 e Å^−3^
Δρ_min_ = −0.14 e Å^−3^

### 

Data collection: *APEX2* (Bruker, 2009 ▶); cell refinement: *SAINT* (Bruker, 2009 ▶); data reduction: *SAINT*; program(s) used to solve structure: *SHELXS97* (Sheldrick, 2008 ▶); program(s) used to refine structure: *SHELXL97* (Sheldrick, 2008 ▶); molecular graphics: *ORTEP-3 for Windows* (Farrugia, 1997 ▶) and *PLATON* (Spek, 2009 ▶); software used to prepare material for publication: *WinGX* (Farrugia, 1999 ▶) and *PLATON*.

## Supplementary Material

Crystal structure: contains datablock(s) global, I. DOI: 10.1107/S1600536811033393/hb6372sup1.cif

Structure factors: contains datablock(s) I. DOI: 10.1107/S1600536811033393/hb6372Isup2.hkl

Supplementary material file. DOI: 10.1107/S1600536811033393/hb6372Isup3.cml

Additional supplementary materials:  crystallographic information; 3D view; checkCIF report

## Figures and Tables

**Table 1:** Hydrogen-bond geometry (Å, °)

*D*—H⋯*A*	*D*—H	H⋯*A*	*D*⋯*A*	*D*—H⋯*A*
O3—H3*A*⋯O1^i^	0.82	1.91	2.723 (2)	169
C13—H13*C*⋯O4	0.96	2.43	3.064 (4)	124

Symmetry code: (i) 
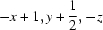
.

**Fig. 1.**
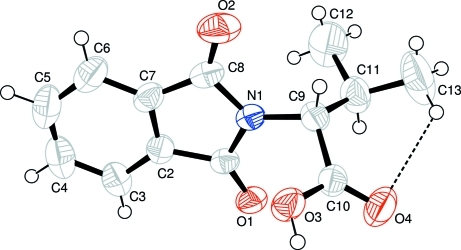
View of the title compound with displacement ellipsoids drawn at the 50% probability level. The dotted line indicates the intramolecular H-bond.

**Fig. 2.**
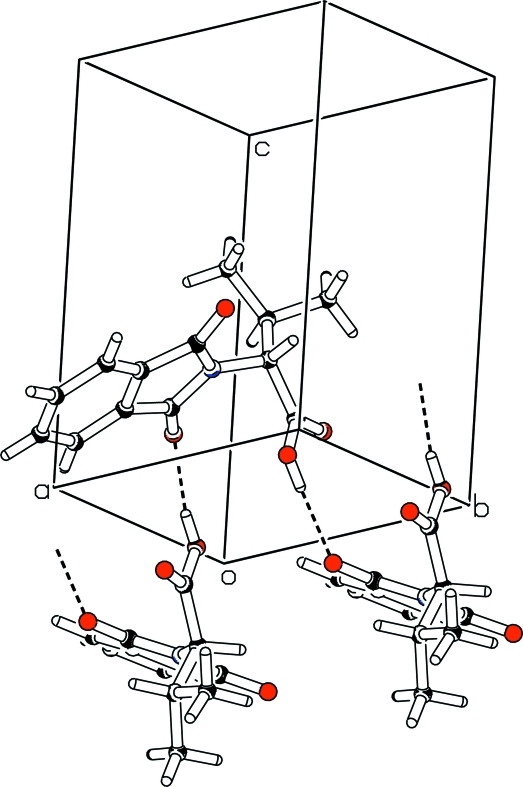
The partial packing, which shows that molecules form one dimensional polymeric network parallel extending along the b axis.

## supplementary crystallographic information

### Comment

The title compound (I, Fig. 1) is being submitted as a part of our research work to synthesize different compounds of phthalic anhydride and various amino acids. In this context, we have reported the crystal structure of (II) *i.e.*, (2*R*)-2-(1,3-dioxoisoindolin-2-yl)-4-(methylsulfanyl)butanoic acid (Raza *et al.*, 2009). The crystal structure of (III) *i.e.*, 2-phthaliminoethanoic acid (Barooah, *et al.*, 2006) has also been published which is related to (I).

In (I), the group A (C1–C8/N1/O1/O2) of 1*H*-isoindole-1,3(2*H*)- dione moiety and the group B (C9/C10/O3/O4) of valine are almost planar with r.m.s. deviations of 0.011 and 0.005 Å, respectively. The propane group C (C11/C12/C13) of valine is of course planar. The dihedral angle between A/B, A/C and B/C is 67.15 (9), 54.87 (30) and 51.52 (22)°, respectively. There exist intramolecular H-bondings of C—H···O type (Table 1, Fig. 1) completing S(6) ring motif (Bernstein *et al.*, 1995). The molecules are stabilized in the form of infinite one dimensional polymeric chains along the *b* axis due to intermolecular hydrogen bonds of the O—H···O type (Table 1, Fig. 2). There does not exist any kind of significant π interaction.

### Experimental

Valine (1.57 g, 13.4 mmol) and phthalic anhydride (2.13 g, 14.38 mmol) were added to a flask with constant stirring at 423 K for 2 h. The reaction mixture was brought to room temperature and the crystalline phthallic anhydride on the walls of the flask were removed. The solid crude product was purified by crystallization from ethanol:water (7:3) that afforded light blue prisms of the title compound (I).

### Refinement

Anomalous dispersion was negligible and Friedel pairs were merged before refinement. The H-atoms were positioned geometrically with (O–H = 0.82, C–H = 0.93–0.98 Å) and were included in the refinement in the riding model approximation, with *U*_iso_(H) = *xU*_eq_(C, O), where *x* = 1.5 for hydroxy & methyl H-atoms and *x* = 1.2 for other H-atoms.

### Crystal data

**Table:**

C_13_H_13_NO_4_	*F*(000) = 260
*M**_r_* = 247.24	*D*_x_ = 1.305 Mg m^−^^3^
Monoclinic, *P*2_1_	Mo *K*α radiation, λ = 0.71073 Å
Hall symbol: P 2yb	Cell parameters from 1267 reflections
*a* = 8.9120 (7) Å	θ = 2.5–27.9°
*b* = 6.3410 (4) Å	µ = 0.10 mm^−^^1^
*c* = 11.8471 (10) Å	*T* = 296 K
β = 109.980 (4)°	Prism, light blue
*V* = 629.20 (8) Å^3^	0.34 × 0.26 × 0.24 mm
*Z* = 2	

### Data collection

**Table:**

Bruker Kappa APEXII CCD diffractometer	1635 independent reflections
Radiation source: fine-focus sealed tube	1267 reflections with *I* > 2σ(*I*)
graphite	*R*_int_ = 0.027
Detector resolution: 7.7 pixels mm^-1^	θ_max_ = 27.9°, θ_min_ = 2.5°
ω scans	*h* = −11→11
Absorption correction: multi-scan (*SADABS*; Bruker, 2005)	*k* = −8→8
*T*_min_ = 0.968, *T*_max_ = 0.978	*l* = −15→15
5969 measured reflections	

### Refinement

**Table:**

Refinement on *F*^2^	Primary atom site location: structure-invariant direct methods
Least-squares matrix: full	Secondary atom site location: difference Fourier map
*R*[*F*^2^ > 2σ(*F*^2^)] = 0.041	Hydrogen site location: inferred from neighbouring sites
*wR*(*F*^2^) = 0.100	H-atom parameters constrained
*S* = 1.05	*w* = 1/[σ^2^(*F*_o_^2^) + (0.0515*P*)^2^ + 0.0165*P*] where *P* = (*F*_o_^2^ + 2*F*_c_^2^)/3
1635 reflections	(Δ/σ)_max_ < 0.001
166 parameters	Δρ_max_ = 0.13 e Å^−^^3^
0 restraints	Δρ_min_ = −0.14 e Å^−^^3^

### Special details

**Table:**

Geometry. Bond distances, angles *etc*. have been calculated using the rounded fractional coordinates. All su's are estimated from the variances of the (full) variance-covariance matrix. The cell e.s.d.'s are taken into account in the estimation of distances, angles and torsion angles
Refinement. Refinement of *F*^2^ against ALL reflections. The weighted *R*-factor *wR* and goodness of fit *S* are based on *F*^2^, conventional *R*-factors *R* are based on *F*, with *F* set to zero for negative *F*^2^. The threshold expression of *F*^2^ > σ(*F*^2^) is used only for calculating *R*-factors(gt) *etc*. and is not relevant to the choice of reflections for refinement. *R*-factors based on *F*^2^ are statistically about twice as large as those based on *F*, and *R*- factors based on ALL data will be even larger.

### Fractional atomic coordinates and isotropic or equivalent isotropic displacement parameters (Å^2^)

**Table:**

	*x*	*y*	*z*	*U*_iso_*/*U*_eq_	
O1	0.63294 (18)	0.2169 (3)	0.16180 (14)	0.0529 (5)	
O2	1.0337 (2)	0.6899 (4)	0.31913 (18)	0.0733 (7)	
O3	0.60132 (17)	0.6706 (3)	0.05589 (13)	0.0577 (6)	
O4	0.4167 (2)	0.6777 (4)	0.14255 (16)	0.0743 (8)	
N1	0.80699 (19)	0.4850 (3)	0.24640 (16)	0.0428 (6)	
C1	0.7654 (3)	0.2944 (3)	0.18945 (19)	0.0411 (7)	
C2	0.9110 (3)	0.2094 (4)	0.17135 (19)	0.0441 (7)	
C3	0.9340 (3)	0.0270 (4)	0.1157 (2)	0.0582 (9)	
C4	1.0854 (4)	−0.0072 (6)	0.1117 (3)	0.0743 (11)	
C5	1.2072 (4)	0.1337 (6)	0.1601 (3)	0.0749 (13)	
C6	1.1836 (3)	0.3191 (5)	0.2150 (3)	0.0651 (10)	
C7	1.0331 (3)	0.3524 (4)	0.2200 (2)	0.0483 (8)	
C8	0.9695 (3)	0.5322 (4)	0.2691 (2)	0.0482 (8)	
C9	0.6940 (3)	0.6354 (4)	0.2664 (2)	0.0475 (7)	
C10	0.5531 (3)	0.6619 (4)	0.1499 (2)	0.0474 (8)	
C11	0.6453 (4)	0.5844 (5)	0.3757 (2)	0.0649 (10)	
C12	0.7868 (4)	0.5100 (9)	0.4803 (3)	0.1015 (18)	
C13	0.5689 (5)	0.7743 (7)	0.4111 (3)	0.113 (2)	
H3	0.85144	−0.06851	0.08244	0.0699*	
H3A	0.52335	0.67521	−0.00594	0.0866*	
H4	1.10506	−0.12888	0.07523	0.0890*	
H5	1.30763	0.10496	0.15619	0.0897*	
H6	1.26560	0.41595	0.24673	0.0782*	
H9	0.74879	0.77181	0.28290	0.0570*	
H11	0.56633	0.47043	0.35343	0.0778*	
H12A	0.87100	0.61231	0.49703	0.1522*	
H12B	0.82370	0.37748	0.46052	0.1522*	
H12C	0.75565	0.49291	0.54970	0.1522*	
H13A	0.64598	0.88579	0.43682	0.1699*	
H13B	0.53183	0.73678	0.47557	0.1699*	
H13C	0.48032	0.82095	0.34336	0.1699*	

### Atomic displacement parameters (Å^2^)

**Table:**

	*U*^11^	*U*^22^	*U*^33^	*U*^12^	*U*^13^	*U*^23^
O1	0.0399 (8)	0.0645 (10)	0.0516 (9)	−0.0117 (8)	0.0123 (7)	−0.0078 (9)
O2	0.0627 (11)	0.0696 (12)	0.0762 (12)	−0.0232 (11)	0.0090 (9)	−0.0166 (12)
O3	0.0446 (9)	0.0857 (14)	0.0411 (8)	0.0087 (10)	0.0125 (6)	0.0097 (10)
O4	0.0454 (10)	0.1094 (17)	0.0700 (12)	0.0175 (12)	0.0223 (8)	0.0094 (13)
N1	0.0363 (10)	0.0467 (10)	0.0405 (9)	0.0011 (9)	0.0067 (7)	−0.0027 (8)
C1	0.0395 (12)	0.0453 (12)	0.0350 (11)	−0.0019 (10)	0.0082 (9)	0.0017 (10)
C2	0.0435 (11)	0.0503 (12)	0.0389 (11)	0.0056 (11)	0.0148 (9)	0.0077 (11)
C3	0.0675 (16)	0.0585 (16)	0.0540 (15)	0.0075 (13)	0.0276 (12)	0.0010 (12)
C4	0.090 (2)	0.075 (2)	0.0713 (19)	0.027 (2)	0.0449 (17)	0.0102 (17)
C5	0.0615 (18)	0.102 (3)	0.0733 (19)	0.0265 (19)	0.0387 (15)	0.024 (2)
C6	0.0426 (14)	0.089 (2)	0.0635 (16)	−0.0001 (14)	0.0179 (12)	0.0150 (16)
C7	0.0373 (12)	0.0646 (16)	0.0406 (12)	0.0017 (11)	0.0102 (9)	0.0093 (11)
C8	0.0397 (12)	0.0545 (15)	0.0425 (12)	−0.0060 (11)	0.0039 (10)	0.0036 (11)
C9	0.0494 (13)	0.0479 (13)	0.0441 (12)	0.0053 (11)	0.0146 (10)	−0.0039 (11)
C10	0.0455 (13)	0.0529 (14)	0.0445 (12)	0.0078 (11)	0.0164 (9)	0.0022 (11)
C11	0.0712 (18)	0.081 (2)	0.0475 (14)	0.0195 (15)	0.0266 (13)	0.0049 (13)
C12	0.104 (3)	0.153 (4)	0.0490 (17)	0.039 (3)	0.0281 (16)	0.029 (2)
C13	0.155 (4)	0.126 (4)	0.076 (2)	0.062 (3)	0.062 (2)	0.003 (2)

### Geometric parameters (Å, °)

**Table:**

O1—C1	1.216 (3)	C9—C10	1.525 (3)
O2—C8	1.203 (3)	C9—C11	1.534 (4)
O3—C10	1.325 (3)	C11—C12	1.510 (5)
O4—C10	1.193 (3)	C11—C13	1.512 (6)
O3—H3A	0.8200	C3—H3	0.9300
N1—C8	1.412 (3)	C4—H4	0.9300
N1—C9	1.464 (3)	C5—H5	0.9300
N1—C1	1.372 (3)	C6—H6	0.9300
C1—C2	1.487 (4)	C9—H9	0.9800
C2—C3	1.381 (4)	C11—H11	0.9800
C2—C7	1.382 (4)	C12—H12A	0.9600
C3—C4	1.383 (5)	C12—H12B	0.9600
C4—C5	1.371 (5)	C12—H12C	0.9600
C5—C6	1.394 (5)	C13—H13A	0.9600
C6—C7	1.379 (4)	C13—H13B	0.9600
C7—C8	1.478 (4)	C13—H13C	0.9600
			
C10—O3—H3A	109.00	C9—C11—C12	111.1 (3)
C1—N1—C9	124.6 (2)	C2—C3—H3	122.00
C8—N1—C9	123.3 (2)	C4—C3—H3	122.00
C1—N1—C8	111.6 (2)	C3—C4—H4	119.00
O1—C1—C2	129.0 (2)	C5—C4—H4	119.00
N1—C1—C2	106.7 (2)	C4—C5—H5	119.00
O1—C1—N1	124.3 (2)	C6—C5—H5	119.00
C1—C2—C7	107.7 (2)	C5—C6—H6	122.00
C3—C2—C7	121.6 (3)	C7—C6—H6	122.00
C1—C2—C3	130.7 (2)	N1—C9—H9	107.00
C2—C3—C4	117.0 (3)	C10—C9—H9	106.00
C3—C4—C5	121.7 (3)	C11—C9—H9	106.00
C4—C5—C6	121.4 (3)	C9—C11—H11	108.00
C5—C6—C7	117.0 (3)	C12—C11—H11	108.00
C2—C7—C8	108.5 (2)	C13—C11—H11	108.00
C6—C7—C8	130.1 (3)	C11—C12—H12A	109.00
C2—C7—C6	121.4 (3)	C11—C12—H12B	109.00
O2—C8—N1	123.7 (2)	C11—C12—H12C	109.00
O2—C8—C7	130.8 (3)	H12A—C12—H12B	109.00
N1—C8—C7	105.5 (2)	H12A—C12—H12C	110.00
N1—C9—C10	108.94 (19)	H12B—C12—H12C	109.00
N1—C9—C11	114.1 (2)	C11—C13—H13A	109.00
C10—C9—C11	113.8 (2)	C11—C13—H13B	109.00
O3—C10—C9	111.2 (2)	C11—C13—H13C	109.00
O4—C10—C9	125.4 (2)	H13A—C13—H13B	109.00
O3—C10—O4	123.4 (2)	H13A—C13—H13C	109.00
C9—C11—C13	110.5 (3)	H13B—C13—H13C	109.00
C12—C11—C13	110.5 (3)		
			
C8—N1—C1—O1	178.8 (2)	C3—C2—C7—C6	0.0 (4)
C8—N1—C1—C2	−0.8 (2)	C3—C2—C7—C8	178.3 (2)
C9—N1—C1—O1	−9.3 (3)	C2—C3—C4—C5	−0.3 (4)
C9—N1—C1—C2	171.12 (19)	C3—C4—C5—C6	−0.4 (5)
C1—N1—C8—O2	−179.3 (2)	C4—C5—C6—C7	1.0 (5)
C1—N1—C8—C7	0.5 (2)	C5—C6—C7—C2	−0.8 (4)
C9—N1—C8—O2	8.7 (4)	C5—C6—C7—C8	−178.6 (3)
C9—N1—C8—C7	−171.57 (19)	C2—C7—C8—O2	179.8 (3)
C1—N1—C9—C10	−46.8 (3)	C2—C7—C8—N1	0.1 (3)
C1—N1—C9—C11	81.6 (3)	C6—C7—C8—O2	−2.2 (5)
C8—N1—C9—C10	124.2 (2)	C6—C7—C8—N1	178.1 (3)
C8—N1—C9—C11	−107.4 (3)	N1—C9—C10—O3	−41.0 (3)
O1—C1—C2—C3	2.6 (4)	N1—C9—C10—O4	140.5 (3)
O1—C1—C2—C7	−178.8 (2)	C11—C9—C10—O3	−169.6 (2)
N1—C1—C2—C3	−177.8 (2)	C11—C9—C10—O4	12.0 (4)
N1—C1—C2—C7	0.8 (2)	N1—C9—C11—C12	40.7 (4)
C1—C2—C3—C4	179.0 (3)	N1—C9—C11—C13	163.8 (3)
C7—C2—C3—C4	0.5 (4)	C10—C9—C11—C12	166.6 (3)
C1—C2—C7—C6	−178.8 (2)	C10—C9—C11—C13	−70.4 (3)
C1—C2—C7—C8	−0.5 (3)		

### Hydrogen-bond geometry (Å, °)

**Table:**

*D*—H···*A*	*D*—H	H···*A*	*D*···*A*	*D*—H···*A*
O3—H3A···O1^i^	0.82	1.91	2.723 (2)	169
C13—H13C···O4	0.96	2.43	3.064 (4)	124

Symmetry codes: (i) −*x*+1, *y*+1/2, −*z*.
